# Creatine Kinase Blockade Disrupts Energy Metabolism and Redox Homeostasis to Suppress Osteosarcoma Progression

**DOI:** 10.3390/ijms262311555

**Published:** 2025-11-28

**Authors:** Shingo Kishi, Rika Sasaki, Rina Fujiwara-Tani, Hitoshi Ohmori, Yi Luo, Kiyomu Fujii, Takamitsu Sasaki, Kei Goto, Yoshihiro Miyagawa, Isao Kawahara, Ryoichi Nishida, Shota Nukaga, Yukiko Nishiguchi, Ruiko Ogata, Kanya Honoki, Hiroki Kuniyasu

**Affiliations:** 1 Department of Molecular Pathology, School of Medicine, Nara Medical University, Kashihara 634-8521, Japan; rika0st1113v726296v@icloud.com (R.S.); rina_fuji@naramed-u.ac.jp (R.F.-T.); brahmus73@hotmail.com (H.O.); lynantong@hotmail.com (Y.L.); toto1999-dreamtheater2006-sms@nifty.com (K.F.); takamitu@fc4.so-net.ne.jp (T.S.); ilgfgtk@gmail.com (K.G.); y.miya1103@gmail.com (Y.M.); isao_kawahara@a011.broada.jp (I.K.); g.m__r1@outlook.jp (R.N.); shota.nukaga@gmail.com (S.N.); yukko10219102@yahoo.co.jp (Y.N.); pkuma.og824@gmail.com (R.O.); 2 Department of Pathology, School of Medicine, Kansai Medical University, Hirakata 573-1010, Japan; 3 Department of Soft Tissue Sarcomas, School of Medicine, Nara Medical University, Kashihara 634-8522, Japan; kahonoki@naramed-u.ac.jp

**Keywords:** creatine kinase B, mitochondrial creatine kinase, phosphorylation signal pathway, osteosarcoma, dinitrofluorobenzen

## Abstract

Osteosarcoma is the most common primary malignant bone tumor in adolescents and young adults; yet survival outcomes have remained stagnated for decades, underscoring the urgent need for new therapeutic strategies. Creatine kinase (CK)—comprising cytosolic CKB and mitochondrial CK—maintains malignant behaviors by supporting high-energy phosphate transfer through the phosphocreatine (pCr) shuttle. Here, we pharmacologically inhibited CK activity in osteosarcoma models and evaluated proliferation, cell death modalities, mitochondrial function, stemness, motility, and tumor behavior. CK blockade consistently suppressed growth and clonogenicity and induced apoptosis as the predominant mode of death. It impaired ATP buffering capacity and disturbed mitochondrial homeostasis, accompanied by reduced expression of stemness-associated markers and diminished migration and invasion. In mouse models, CK inhibition significantly restrained tumor progression and dissemination. These results indicate that disabling the CK-pCr energy-buffering system reprograms cellular energetics toward apoptosis and less aggressive phenotypes. Our findings support targeting the CK pathway as a tractable metabolic vulnerability and a rational partner for cytotoxic regimens, with pathway-specific signaling alterations representing downstream consequences of central energetic collapse.

## 1. Introduction

Osteosarcoma (OS) is the most common primary malignant bone tumor, occurring predominantly in adolescents and young adults, and arising most frequently in the metaphyseal regions of long bones [1,2]. Standard chemotherapy for pediatric and adolescent and young adult (AYA) patients comprises high-dose methotrexate, doxorubicin, and cisplatin (MAP), whereas older adults (≥40 years) are generally treated with doxorubicin plus cisplatin, with or without ifosfamide, to avoid methotrexate [3,4]. Although long-term survival for localized disease approaches 60–70%, outcomes for metastatic or relapsed OS remain dismal. Importantly, survival rates for localized OS have stagnated since the 1980s due to the absence of new effective therapeutic options [4], underscoring an urgent need to identify novel molecular targets.

Recent studies have highlighted that osteosarcoma exhibits marked metabolic plasticity; however, the mechanisms that stabilize its energy balance remain poorly defined. A hallmark of malignant metabolism is the Warburg effect, wherein cancer cells preferentially utilize aerobic glycolysis despite intact mitochondrial function [5]. This configuration supports biosynthesis and redox capacity yet leaves cells vulnerable to fluctuations in ATP demand. The creatine kinase (CK)–phosphocreatine (pCr) shuttle is a key ATP-buffering system that rapidly regenerates ATP and integrates glycolytic and mitochondrial energy fluxes. Disruption of this CK-dependent circuit is therefore predicted to critically impair OS growth and survival, although its precise contribution remains unsolved.

Creatine kinases (CKs) catalyze the reversible transfer of high-energy phosphate groups between ATP and creatine, maintaining cellular energy homeostasis (Figure S1). Cytosolic CKB (brain-type CK) and mitochondrial MTCK (CKMT1/2) have emerged as important regulators in cancer metabolism [6,7,8].

CKB supports ATP supply to energy-demanding sites and contributes to cytoskeletal remodeling, migration, and invasion. It is frequently upregulated in multiple malignancies, including breast and colorectal carcinomas. Inhibition or miRNA-mediated suppression of CKB reduces proliferation, invasion, epithelial–mesenchymal transition, and metastatic potential, highlighting its therapeutic relevance [9,10,11,12]. By contrast, normal cells rely more on alternative pathways and are less dependent on CKB.

MTCK, located in the mitochondrial intermembrane space, converts ATP to pCr, which diffuses into the cytosol while ADP is recycled back into the mitochondria [13,14]. This mechanism fuels energy-consuming processes, stabilizes mitochondrial redox balance, and prevents excessive reactive oxygen species (ROS) generation (Figure S1). MTCK inhibition or deficiency leads to the collapse of the ATP supply, oxidative stress, mitochondrial permeability transition pore (mPTP), and apoptosis [15,16]. Although MTCK is indispensable for tumor cell survival, it is also critical in high-energy tissues such as the myocardium and neurons, raising concerns regarding potential systemic toxicity [17,18].

Multiple strategies targeting CKs have been explored. Cyclocreatine (CyC) shows antitumor activity in vitro and in vivo [19,20,21]. β-Guanidinopropionic acid (β-GPA) depletes pCr and reduces tumor burden [22], and miRNAs such as \miR-483-5p and miR-551a suppress CKB to inhibit metastasis [9]. Mitochondrial CK inhibition increases ROS and induces apoptosis [15]. Pharmacological blockade of the creatine shuttle using 2,4-dinitro-1-fluorobenzene (DNFB) suppresses colorectal cancer growth and metastatic ability in vitro [23], and covalent CK inhibitors targeting catalytic cysteine residues demonstrate antitumor effects in glioblastoma models [24,25]. However, systemic CK inhibition causes cardiac and skeletal toxicity, limiting clinical translation [26,27]. Conversely, the creatine transporter (SLC6A8) inhibitor RGX-202 is in early-phase clinical evaluation [28].

DNFB, originally introduced by Sanger as a derivatization reagent, is an electrophilic arylating compound that forms dinitrophenyl (DNP) adducts with protein nucleophiles such as cysteine and lysine. Due to this broad reactivity, high-dose repetitive topical DNFB can act as a tumor-promoting agent in initiated mouse skin models [29]. Therefore, in the present study, DNFB was used solely as an in vitro tool compound to acutely inactivate CK; our therapeutic concept focuses on targeting the CK–pCr axis rather than on clinical use of DNFB itself.

Although CK activity has been implicated in cell proliferation and its inhibition exerts antitumor effects [30,31], the functional role of CK isoforms in OS remains poorly defined compared with other malignancies, such as colorectal, pancreatic, breast, and hepatocellular carcinomas [32]. Here, we investigated the effects of CK inhibition in OS cells and xenograft models to establish CKB and MTCK as potential therapeutic targets.

## 2. Results

### 2.1. Effects of CK Inhibition on OS Cells

**Figure 1:**
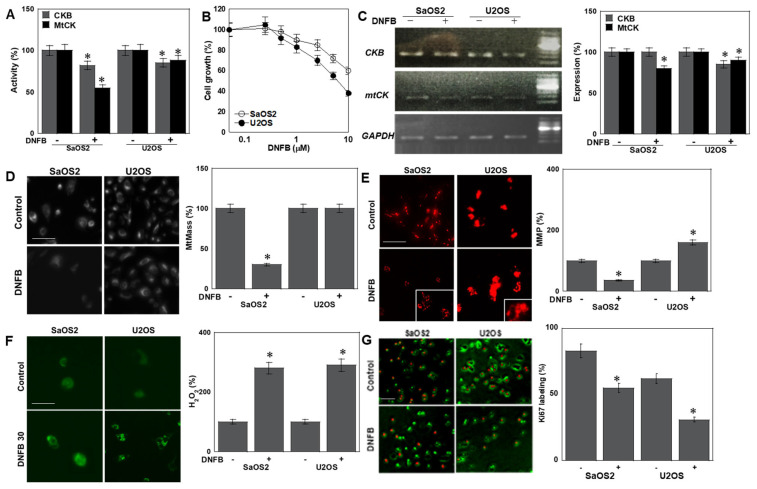
Effects of DNFB-mediated CK inhibition on OS cells. (**A**,**B**) DNFB suppressed CKB/MTCK enzymatic activity and reduced cell proliferation in a dose-dependent manner. (**C**) mRNA expression of CKB and MTCK following DNFB treatment (quantified by RT-PCR). (**D**–**F**) Effects of DNFB on mitochondria: mitochondrial parameters: mitochondrial mass (MtMass), MMP (**E**). Inset, high magnificent image, and mitochondrial H_2_O_2_ production, assessed by MitoTracker Green, TMRE, and MitoSOX fluorescence, respectively. Scale bar, 50 μm. (**G**) Labeling index of proliferation index quantified by Ki67 immunofluorescence * *p* < 0.05 vs. DNFB(−). Error bars: standard deviation of three independent trials. Statistical differences were calculated using ordinary ANOVA with Bonferroni correction. ANOVA, analysis of variance; DNFB, dinitrofluorobenzene; CK, creatine kinase; OS, osteosarcoma; CKB, creatine kinase B; MTCK, mitochondrial creatine kinase; mtMass, mitochondrial mass; MMP, mitochondrial membrane potential. Sphere formation assays revealed that DNFB significantly impaired the clonogenic potential in both cell lines (Figure 2A), accompanied by reduced expression of stemness markers such as *POU Class 5 Homeobox 1* (Oct3) and *Nestin* (Figure 2B).

Treatment with DNFB, a pharmacologic CK inhibitor, suppressed the enzymatic activity of both CKB and MTCK, resulting in a dose-dependent reduction in cell proliferation (Figure 1A,B). Additional OS cell lines (HOS and MG63) similarly demonstrated DNFB dose-dependent growth inhibition (Supplementary Figure S2A). DNFB exposure also decreased CKB and MTCK mRNA levels (Figure 1C). Mitochondrial mass (MtMass) was reduced in SaOS2 and HOS cells but remained unchanged in U2OS and MG63 cells (Figure 1D, Supplementary Figure S2B,D). Mitochondrial membrane potential (MMP) decreased in SaOS2 cells yet paradoxically increased in U2OS cells (Figure 1E, Supplementary Figure S2C,D). In contrast, mitochondrial H_2_O_2_ production increased in both lines (Figure 1F). The proliferative index, measured by Ki67 staining, was markedly reduced following CK inhibition (Figure 1G). To investigate metabolic differences underlying variable DNFB responses, Seahorse flux analysis was performed, revealing an OXPHOS-dominant phenotype in SaOS2 and HOS, whereas U2OS and MG63 relied more heavily on glycolysis (Supplementary Figure S2E).

**Figure 2:**
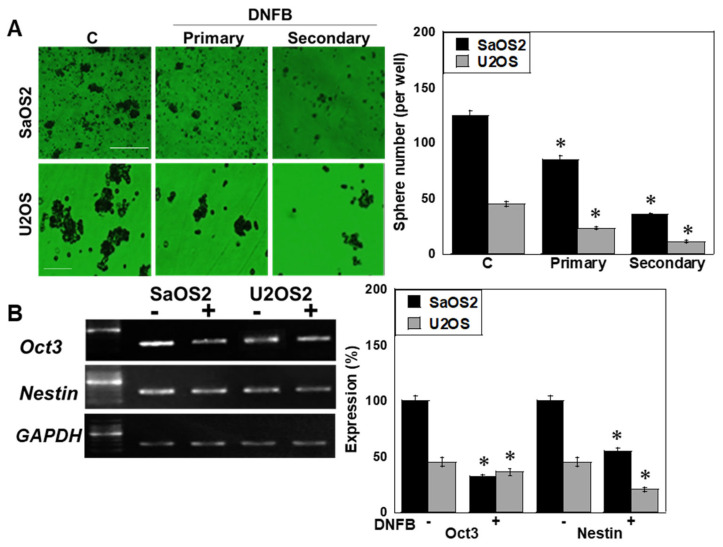
Effects of CK inhibition on stemness. (**A**) Sphere-forming capacity in SaOS2 and U2OS cells following DNFB treatment. Images were taken with a phase-contrast microscope. Scale bar, 100 μm. (**B**) Expression of stemness markers *Oct3* and *Nestin* assessed by RT-PCR. * *p* < 0.05 vs. C or DNFB(−). Error bars: standard deviation of three independent trials. Statistical differences were calculated using ordinary ANOVA with Bonferroni correction. ANOVA, analysis of variance; DNFB, dinitrofluorobenzene; CK, creatine kinase; OCT3, POU Class 5 homeobox 1; ACTB, β-actin.

### 2.2. Effects of CK Inhibition on Cell Death

**Figure 3:**
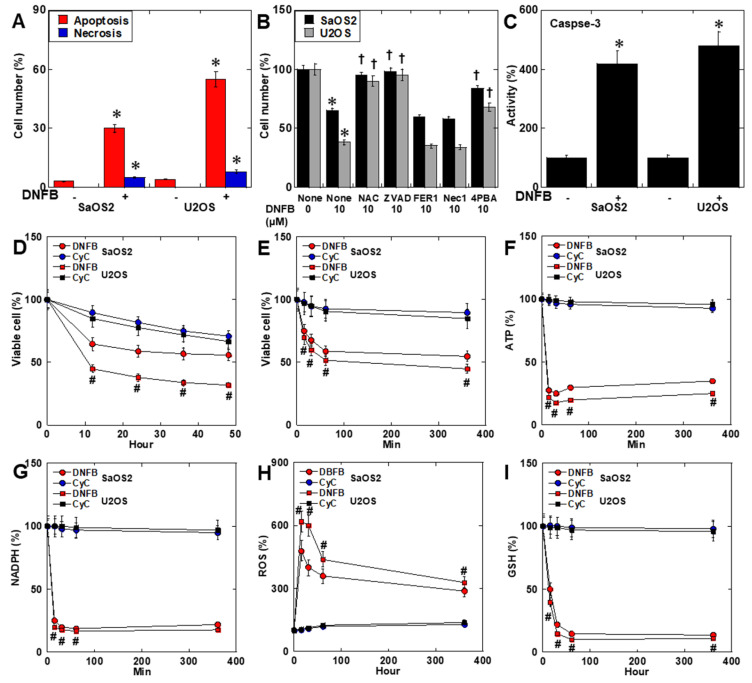
DNFB-induced cell death. (**A**) Apoptosis and necrosis evaluated using by Cell Meter^TM^ flowcytometric cell death detection kit. (**B**) Cell death rescue assay using: NAC, Z-VAD-FMK, Fer-1, and 4PBA. (**C**) Caspase-3 activity. (**D**,**E**) Time-course comparison of viability loss induced by DNFB and cyclocreatine (CyC, 500 μM). (**F**–**I**) Early decrease in ATP and NADPH levels, transient increase in ROS (4HNE), and progressive GSH depletion, evaluated by ELISA kits. * *p* < 0.05 vs. DNFB(−) or None/DNFB(−) or CyC. ^†^ *p* < 0.05 vs. None/DNFB (10), ^#^ *p* < 0.05 vs. CyC (both cell types). Data represent mean ± SD of three independent experiments. Statistical differences were calculated using ordinary ANOVA with Bonferroni correction. ANOVA, analysis of variance; DNFB, dinitrofluorobenzene; NAC, N-acetylcysteine; ZVAD, Z-VAD-FMK; FER1, ferrostatin-1; NEC1, necrostatin-1; 4PBA, 4-Phenylbutyric acid; CyC, cyclocreatine; NADPH, nicotinamide adenine dinucleotide phosphate; GSH, glutathione; 4HNE, 4-hydroxynonenal.

DNFB treatment induced apoptosis in both SaOS2 and U2OS cells, with only a modest increase in necrosis (Figure 3A). Cell death was attenuated by the antioxidant N-acetylcysteine (NAC) and the apoptosis inhibitor Z-VAD, and partially by the ER stress inhibitor 4-PBA, but not by inhibitors of ferroptosis (Fer-1) (Figure 3B). Caspase-3 activity was significantly increased following DNFB exposure (Figure 3C). Time-course analysis revealed rapid loss of viability within 60 min after DNFB treatment, occurring more rapidly than with the creatine analog CyC (Figure 3D,E). ATP and NADPH levels sharply declined during this early phase (Figure 3F,G), while ROS levels peaked at 15 min and gradually declined thereafter in parallel with GSH depletion (Figure 3H,I).

### 2.3. Effects of CK Inhibition on Phosphorylation Signaling

**Figure 4:**
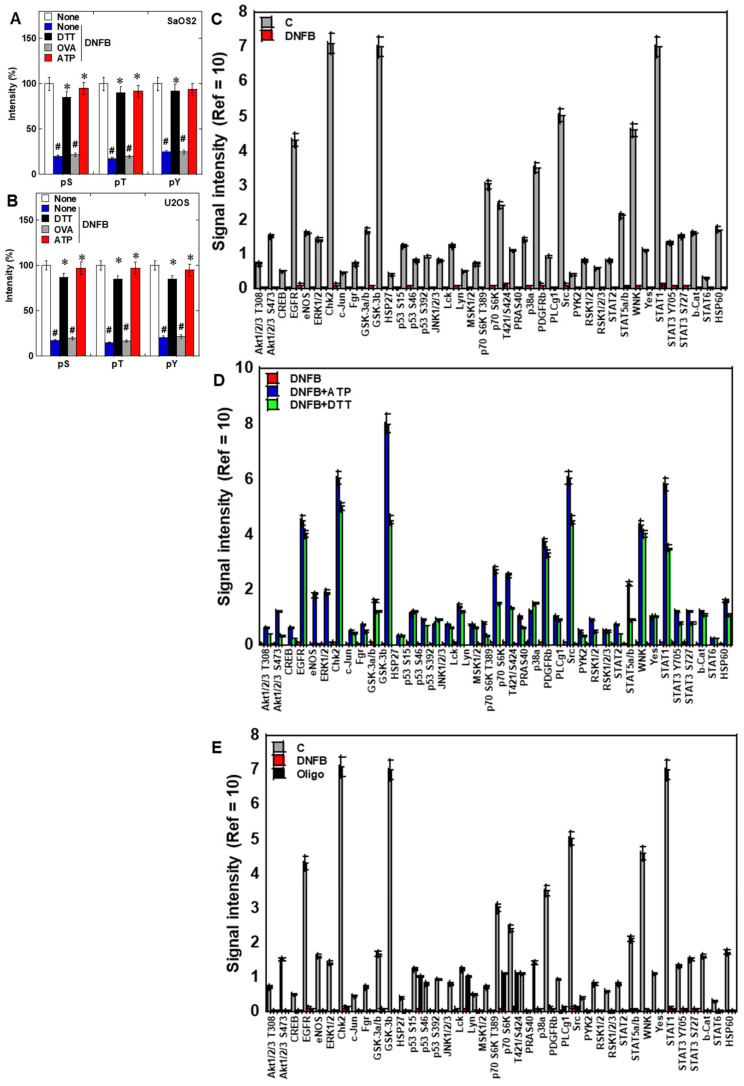
CK inhibition suppresses phosphorylation signaling networks. (**A**,**B**) DNFB caused a global reduction in serine, threonine, and tyrosine residues, restored by ATP (1 mM) or DTT (0.5 mM). Evaluated by dot blot analysis of proteins. (**C**) Proteome Profiler Phospho-Kinase array revealed broad suppression of 39 signaling proteins. (**D**,**E**) ATP (1 mM) or DTT (0.5 mM) restored phosphorylation, whereas oligomycin (0.5 μM) mimicked DNFB effects. ^#^ *p* < 0.05 vs. DNFB(−) or * *p* < 0.05 vs. None/DNFB(+). Data represent mean ± SD of three independent experiments. Statistical differences were calculated using ordinary ANOVA with Bonferroni correction. ANOVA, analysis of variance; DNFB, dinitrofluorobenzene; pS, phosphoserine; pT, phosphothreonine; pY, phosphotyrosine; DTT, dithiothreitol; OVA, orthovanadate; oligo, oligomycin.

Consistent with our prior report of global suppression phosphorylation suppression following DNFB exposure [23], pan-phosphorylation of serine, threonine, and tyrosine residues decreased by approximately 20% in both OS lines (Figure 4A,B). This reduction was restored by dithiothreitol (DTT) or ATP supplementation but not by phosphatase inhibition with pervanadate. Phospho-protein array profiling demonstrated broad downregulation of 39 signaling proteins, including Akt, extracellular signal-regulated kinase (ERK) 1/2, and signal transducer and activator of transcription (STAT)-3 (Figure 4C). ATP or DTT treatment rescued the phosphorylation, whereas oligomycin recapitulated DNFB effect (Figure 4D,E). Western blotting confirmed that DNFB reduced phosphorylation of Akt (Ser473), ERK1/2, and STAT3 (Tyr705) without altering total protein levels, and ATP supplementation restored phosphorylation (Supplementary Figure S3).

### 2.4. Isoform-Specific Effects of CK Knockdown

**Figure 5:**
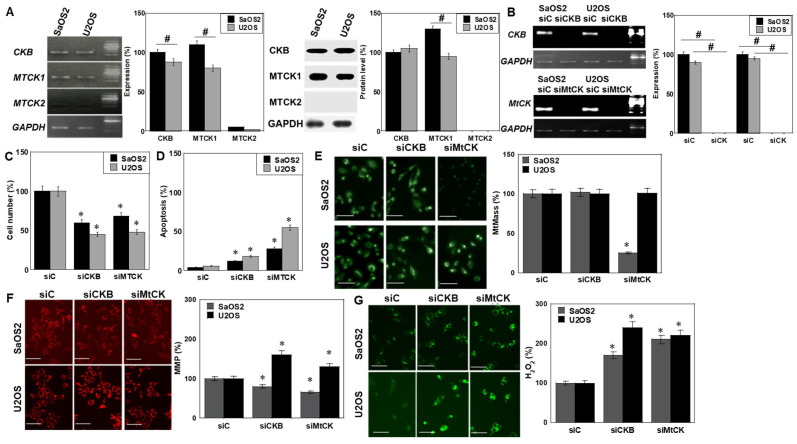
Isoform-specific effects of CK knockdown. (**A**) Basal expression of CK isoforms of mRNA (left, RT-PCR) and protein (right, Western blot). (**B**) Confirmation of siRNA knockdown efficiency, evaluated by RT-PCR. (**C**) Both CKB and MTCK KD reduced proliferation. (**D**) Apoptosis induction was more pronounced after MTCK KD. (**E**,**F**) Effect of KD on MtMass and MMP. (**G**) Mitochondrial H_2_O_2_ generation. Data represent mean ± SD of three independent experiments. * *p* < 0.05 vs. ^#^ p < 0.05. siC. Statistical analysis: one-way ANOVA with Bonferroni correction. ANOVA, analysis of variance; KD, knockdown; si, small interfering RNA; siC, control siRNA; siCKB, CKB siRNA; siMTCK, MTCK1 siRNA; MtMass, mitochondrial mass.

MTCK2 expression was lower than that of CKB and MTCK1 at both mRNA and protein levels (Figure 5A). Knockdown (KD) of either CKB or MTCK1 suppressed cell proliferation in (Figure 5B,C). Apoptosis induction was more pronounced in MTCK1 KD (Figure 5D). In SaOS2 cells, MtMass decreased following MTCK1 KD. MMP was reduced by both KDs in SaOS2 but paradoxically increased in U2OS (Figure 5E,F). Both KDs increased mitochondrial H_2_O_2_ levels (Figure 5G).

### 2.5. CK Specificity of DNFB

Because DNFB is a broadly reactive electrophile, off-target effects could not be excluded. To directly assess CK dependence, we conducted epistasis experiments combining CK ND with DNFB (Supplementary Figure S4). Dual KD of CKB and MTCK1 markedly attenuated DNFB-induced cytotoxicity and apoptosis, supporting CK inhibition as a major mechanism of DNFB action. Differential DNFB responses in multiple OS cell lines were consistent with their distinct metabolic phenotypes (Supplementary Figure S2). These findings demonstrate mechanistic convergence between DNFB and CK KD.

### 2.6. Effects of CK Inhibition In Vivo

**Figure 6:**
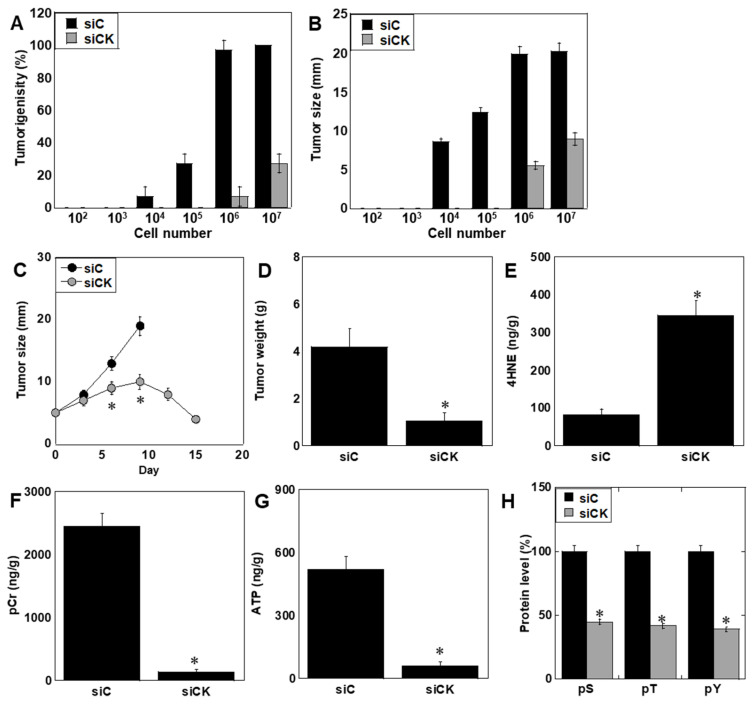
In vivo effects of CK knockdown. Ten mice per group were examined. (**A**) Limiting dilution assay of tumorigenicity. (**B**) Tumor growth was suppressed in CK KD xenografts. (**C**) Intratumoral siRNA-liposome injection suppressed tumor growth and induced regression. (**D**) Final tumor weight. (**E**–**G**) Increased 4-HNE, decreased phosphocreatine, and ATP in CK KD tumors. (**H**) Reduced global phosphorylation levels. Evaluated by dot blot analysis of protein. * *p* < 0.05 vs. siC. Data represent mean ± SD of 10 mice. Statistical differences were calculated using ordinary ANOVA with Bonferroni correction. ANOVA, analysis of variance; KD, knockdown; si, small interfering RNA; siC, control siRNA; siCK, siCKB + siMTCK1; 4HNE, 4-hydroxynonenal; pCr, phosphocreatine; pS, phosphoserine; pT, phosphothreonine; pY, phosphotyrosine.

Because DNFB is rapidly inactivated in vivo, xenograft experiments were performed using CK-KD cells. Tumor-initiating capacity was markedly reduced: while control SaOS2 cells formed tumors from injections at as low as 10^4^ cells, CK KD cells required ≥10^6^ cells (Figure 6A). Tumor growth was significantly suppressed in CK KD groups (Figure 6B), and intratumoral siRNA-liposome injection induced tumor regression after day 9 (Figure 6C). CK KD significantly reduced final tumor weight and increased oxidative stress markers (4-HNE), while phosphocreatine, ATP, and global phosphorylation levels were markedly reduced (Figure 6D–H).

Toxicity assessments revealed no significant differences in body weight, organ weight, or serum biochemical parameters (ALT, AST, BUN) between groups (Supplementary Figure S5A–C). MSC-derived osteoblast-like cells (MSC-OB) displayed substantially greater resistance to DNFB than OS cells (IC50: SaOS2 18.2 μM, U2OS 9.3 μM, MSC-OB 95 μM), indicating a potential therapeutic window (Supplementary Figure S5D).

**Figure 7:**
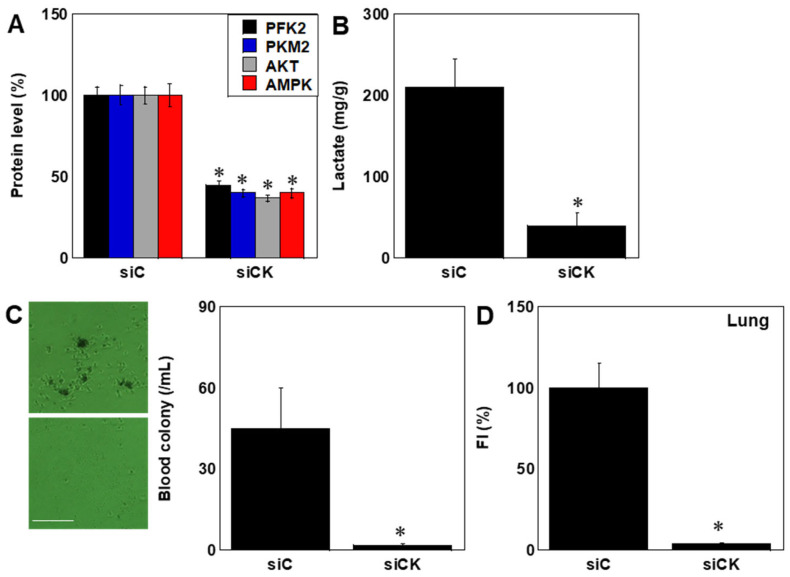
Effects of CK KD on glycolysis and metastasis. (**A**) Expression of glycolysis-related proteins (PFK2, PKM2, Akt, AMPK) measured by ELISA (**B**) Lactate production. (**C**) Circulating tumor cells at 12 h post-tail vein injection. Images were taken with a phase-contrast microscope. Each experimental group consisted of 10 mice. Scale bar, 100 μm. (**D**) Pulmonary colonization of fluorescently labeled OS cells. * *p* < 0.05 vs. siC. Data represent mean ± SD of 10 mice. Statistical differences were calculated using ordinary ANOVA with Bonferroni correction. ANOVA, analysis of variance; KD, knockdown; si, small interfering RNA; siC, control siRNA; siCK, siCKB + siMTCK1; PKM2, pyruvate kinase M2; PFK2, 6-phosphofructo-2-kinase/fructose-2,6-bisphosphatase 2; AMPK, 5′-AMP-activated protein kinase; FI, fluorescent intensity.

Glycolysis-related enzyme expression and lactate production were also reduced (Figure 7A,B). CK KD preconditioning reduced circulating tumor cells 12 h after tail vein injection and significantly decreased pulmonary colonization by fluorescent OS cells (Figure 7C,D). No significant differences in organ weights were observed between groups (supplementary Figure S5B).

## 3. Discussion

In this study, we comprehensively evaluated the effects of creatine kinase (CK) inhibition on OS models in vitro and in vivo. CK blockade suppressed cell proliferation, induced ROS-mediated apoptosis, impaired mitochondrial homeostasis, disrupted phosphorylation signaling, diminished stemness, and reduced both tumorigenicity and metastatic potential. These findings highlight the CK–phosphocreatine (PCr) energy-buffering system as a metabolic vulnerability in OS.

DNFB rapidly inhibited the activities of CKB and MTCK, causing an acute reduction in ATP and NADPH within 1 h. This early energetic collapse was accompanied by alterations in mitochondrial membrane potential and increased ROS production, ultimately leading to apoptosis. This effect was attenuated by the antioxidant NAC, indicating that ROS is a central mediator of DNFB-induced cell death. MTCK protects against mitochondrial ROS by facilitating pCr cycling [33]; thus, its inhibition disrupts redox balance, with minimal contribution from necrosis or ferroptosis. These findings support the concept that CK isoforms are essential not only for ATP buffering but also for oxidative stress resistance in OS cells. Previous studies have linked CKB to oncogenic signaling via suppression of the p53/p21/BAX axis and enhancement of BCL2 and MDM2 [29], while MTCK2 has been identified as a marker of poor-prognosis metastasis [34]. Our results provide mechanistic insights into how inhibition of these isoforms promotes ROS-dependent apoptosis.

Distinct mitochondrial responses were observed among OS cell lines: mitochondrial mass and membrane potential decreased in SaOS2 and HOS but increased in U2OS and MG63 cells. Because MTCK interacts with the voltage-dependent anion channel (VDAC) to maintain mitochondrial electrochemical stability [35], differential MTCK sensitivity to DNFB may contribute to these phenotypes. Seahorse analysis demonstrated that SaOS2 and HOS rely predominantly on oxidative phosphorylation (OXPHOS), whereas U2OS and MG63 favor glycolysis. Thus, MTCK inhibition in OXPHOS-dependent lines may trigger mitochondrial dysfunction and apoptosis, whereas glycolytic lines may undergo apoptosis due to ROS-driven hyperpolarization. These trends were reproduced using siRNA-mediated CK knockdown, in which MTCK1 depletion caused more pronounced mitochondrial dysfunction and oxidative stress. MTCK1 and MTCK2 have also been linked to mitochondrial architecture through polyamine metabolism [36], consistent with our findings.

DNFB broadly suppressed the phosphorylation of serine, threonine, and tyrosine residues, and reduced activation of key survival pathways, including Akt, ERK, and STAT3. These effects were reversed by ATP or DTT, but not by phosphatase inhibition, and were recapitulated by oligomycin, indicating that ATP depletion and oxidative stress are the primary mechanisms. Rapid loss of ATP and GSH likely prevents compensatory responses, explaining the more pronounced effects compared to cyclocreatine. Additionally, GSH depletion can enhance CK thiolation and inactivation [37], potentially reinforcing the energy failure cycle. CK knockdown reduced glycolysis-related enzyme expression and lactate production, indicating that glycolysis does not compensate for impaired mitochondrial function. MTCK1 reportedly promotes HK2-dependent glycolysis via JNK-MAPK/JUN signaling [38], consistent with our observation of dual suppression of OXPHOS and glycolysis. This metabolic “quiescence” aligns with reduced stemness, impaired pulmonary colonization, and reduced circulating tumor cells. CKMT1 regulates trophoblast differentiation [39], and phosphocreatine prevents stemness loss by blocking BRD2 ubiquitination [40]. Furthermore, miR-483 and miR-551a suppress CKB to inhibit metastasis [41]. As reviewed by Du et al. [42], redox-sensitive transcription factors such as Nrf2, HIF-1α, FOXO, and STAT3 are key regulators of cancer stemness. Our findings support a framework in which disruption of the CK–pCr energy-buffering system leads to ATP depletion and redox imbalance, attenuating these pro-stemness pathways in OS.

DNFB, used in this study to inhibit CK, has been reported to inactivate enzymes such as fructose diphosphatase through reactions with amines/thiols [43] or cysteine residues [44]. Given that DNFB is a broadly reactive electrophile, off-target effects must be considered. However, dual knockdown of CKB and MTCK1 markedly attenuated DNFB-induced cytotoxicity and apoptosis, supporting CK inhibition as its major mechanism. While DNFB cannot be used clinically due to rapid inactivation and sensitizing/tumor-promoting potential following chronic dermal exposure, our data validate CK as a molecular target and justify the development of selective CK inhibitors or SLC6A8 inhibitors such as RGX-202 for future translational studies [24,25].

Importantly, CK inhibition demonstrated an acceptable safety profile: CK-KD xenografts showed no significant toxicity in body weight, organ weight, or serum chemistry, and MSC-derived osteoblast-like cells were substantially more resistant to DNFB than OS cells, suggesting a therapeutic window. Long-term evaluation in myocardium and neural tissue—high-CK-demand sites—will be needed prior to clinical translation.

Recent investigations support therapeutic targeting of mitochondrial metabolism in OS. OS stem-like subpopulations co-activate glycolysis and OXPHOS and depend on OXPHOS for progression [45,46], and Complex I inhibition has demonstrated efficacy in OS models and early clinical trials [47,48]. Within this framework, the CK–PCr axis represents a complementary metabolic node that integrates mitochondrial output with cytosolic energy demand. Combining CK axis inhibition with mitochondrial inhibitors or redox-modulating therapy may represent a rational strategy for targeting aggressive OS [49,50,51].

Taken together, our findings establish CK-dependent ATP buffering as a metabolic vulnerability in osteosarcoma. Although DNFB was used solely as a tool compound, convergence between DNFB and CK knockdown strongly supports CK inhibition as a rational therapeutic strategy [52]. Future efforts should prioritize the development of selective CK inhibitors or SLC6A8 transport inhibitors such as RGX-202 [49], and the integration of CK-targeted approaches with metabolic or redox-modulating therapies. This metabolism-anchored perspective may facilitate the development of effective treatments for refractory OS.

## 4. Materials and Methods

### 4.1. Cell Culture

Human OS cell lines SaOS2, U2OS, HOS, MG63, and human bone marrow-derived mesenchymal stem cell line (hMSC, PCS-500-01) were purchased from the American Type Culture Collection (ATCC; Rockville, MD, USA). Cells were cultured in Dulbecco’s Modified Eagle Medium (DMEM; Nacalai Tesque, Kyoto, Japan) supplemented with 10% fetal bovine serum (Nichirei, Tokyo, Japan) and 50 U/mL penicillin/streptomycin (Nacalai Tesque) under 5% CO_2_ at 37 °C. Cyclocreatin (CyC, 500 μM, Selleck, Yokohama, Japan), oligomycin (Oligo, 0.5 μM, Selleck), ATP (1 mM, WAKO, Osaka, Japan), and dithiothreitol (DTT, 0.5 mM, WAKO) were obtained from the indicated suppliers.

For osteoblastic differentiation, hMSCs were cultured in MesenCult Osteogenic Differentiation Basal Medium supplemented with MesenCult Osteogenic Diff 5× Supplement (Veritas, Tokyo, Japan) according to the manufacturer’s protocol.

DNFB (Wako, Osaka, Japan) was dissolved in DMSO and used at 5–20 μM, 48 h for exclusively in vitro. This range was selected based on previous studies in colorectal cancer demonstrating that 10 μM DNFB inhibits CK activity and proliferation without causing extensive necrosis in non-tumor cells [23]. DNFB was not administered systemically in vivo because of rapid protein binding and its reported tumor-promoting effects when repeatedly applied at high-dose dermal doses in initiated mouse skin. All in vivo studies employed CK inhibition via siRNA or intratumoral liposomal delivery.

### 4.2. Cell Viability Assay

Cells (1 × 10^4^ cells/well) were seeded in 96-well plates, cultured overnight, and treated with DNFB (5–20 μM) for 48 h. Viability was measured by MTS tetrazolium assay (Sigma-Aldrich Inc., St. Louis, MO, USA), as previously described [53].

### 4.3. Cell Death and Apoptosis

Apoptosis and necrosis were evaluated using Cell Meter™ Apoptotic and Necrotic Multiplexing Detection Kit (#22843, AAT Bioquest, Pleasanton, CA, USA). Cells were harvested after treatment, washed twice with cold PBS, and resuspended in 1× binding buffer at a concentration of 1 × 10^6^ cells/mL. Loss of plasma membrane integrity, as demonstrated by the ability of a membrane-impermeable DNA Nuclear Green™ DCS1 (Ex/Em = 490/525 nm) to label the nucleus, represents a straightforward approach to demonstrate late-stage apoptosis and necrosis. In addition, this kit also provides a live cell cytoplasm labeling dye, CytoCalcein™ Violet 450 (Ex/Em = 405/450 nm), for labeling living cell cytoplasm. Samples were analyzed within 1 h using a flow cytometer (BD FACSCanto II, BD Biosciences) equipped with Analyze cells with a flow cytometer with 660/20 nm (for apotosis-APC channel), 530/30 nm (for necrosis-FITC channel) and 450/40 nm emission filter (for healthy cells-Pacific Blue channel) A total of 10,000 events were collected for each sample.

### 4.4. Cytoimmunochemistry

Cells grown on glass slides (Nunc, Thermo Fisher, Tokyo, Japan) were fixed with 4% paraformaldehyde (4 °C, 1 h) and permeabilized with Triton-X100 (0.2% in PBS, 4 °C, 10 min). Ki67 immunostaining was performed using anti-Ki67 antibody (Abcam, Waltham, MA, USA) labeled with Cyanine 5 dye (AAT Bioquest, Pleasanton, CA, USA). Ki67 labeling index was calculated by counting 500 cells using a BZ-X710 fluorescence microscope (KEYENCE, Osaka, Japan).

### 4.5. Cell Death Rescue Assay

OS cells were treated with DNFB (10 μM for 48 h) in the presence or absence of the following inhibitors: NAC (WAKO), Z-VAD-FMK (ZVAD, 20 µM; Santa Cruz Biotechnology, Santa Cruz, CA, USA), ferrostatin-1 (FER1, 2 µM; Cayman Chemicals, Ann Arbor, MI, USA), necrostatin-1 (NEC1, Selleck), 4-phenylbutyric acid (4PBA, Tokyo Chemical Institute, Tokyo, Japan). Viability was assessed by MTS assay.

### 4.6. Sphere Formation Assay

Cells (1 × 10^3^) were seeded in bacteriological 35-mm dishes (Corning, NY, USA) containing 3D Tumorsphere Medium XF (Sigma-Aldrich). After 5 days, spheres were dissociated with Accutase (Innovative Cell Technologies) and replated (1 × 10^3^ cells/mL) in ultra-low attachment 6-well plates (Corning). Spheres were imaged on Days 5 and 10, and quantified using ImageJ software (version 1.54, NIH, Bethesda, MD, USA).

### 4.7. Reverse Transcription-Polymerase Chain Reaction (RT-PCR)

Total RNA (0.5 µg) was extracted using the RNeasy Kit (Qiagen, Germantown, MD, USA). Primers (Table 1) were designed using NCBI Primer-BLAST (version 2.5.0) and validated with Primer3Plus to minimize secondary structure and dimer formation. RT-PCR was performed according to the manufacturer’s protocol, and products were electrophoresed on 2% agarose gels stained with ethidium bromide. DNase digestion was not performed, as the purpose was to analyze total cellular RNA rather than extracellular contaminating DNA.

### 4.8. Mitochondrial Imaging

Cells were seeded in 6-well plates (1 × 10^5^ cells/well), treated with or without DNFB (10 µM for 48 h), and stained with dihydrorhodamine 123 (DHR123; 10 mM for mitochondrial H_2_O_2_), tetramethylrhodamine ethyl ester (TMRE, 0.5 µM; for mitochondrial membrane potential, MMP), and MitoGreen (200 µM; for mitochondrial mass, MtMass) in the dark at 37 °C for 30 min. Fluorescence images were acquired using BZ-X700 fluorescence microscope (KEYENCE). Quantification of signal intensities was performed using ImageJ software (version 1.54, NIH, Bethesda, MD, USA).

### 4.9. Protein Extraction

Whole-cell protein lysates were prepared using RIPA buffer supplemented with 0.1% sodium dodecyl sulfate (SDS) (Thermo Fisher Scientific, Tokyo, Japan), following our previously published protocol [53]. Samples were stored at −80 °C until use. Protein concentrations were quantified using the Protein Assay Rapid Kit (Wako Pure Chemical Industries, Osaka, Japan).

### 4.10. Enzyme-Linked Immunosorbent Assay

Enzyme-linked immunosorbent assay (ELISA) kits were used to measure the protein levels and enzyme activities from the extracted samples according to manufacturer’s instructions (Table 1). All assays were performed using whole-cell lysates.

### 4.11. Phosphorylated Protein Profiling

Phospho-protein profiling was performed using the Proteome Profiler Human Phospho-Kinase Array Kit (ARY003C, R&D Systems, Minneapolis, MN, USA) according to the manufacturer’s instructions. This membrane-based sandwich immunoassay uses immobilized capture antibodies spotted in duplicate on nitrocellulose membranes to detect phosphorylated signaling proteins. Captured proteins were detected using biotinylated secondary antibodies and chemiluminescent substrate. Signal intensity was proportional to relative phosphorylation levels.

### 4.12. Immunoblot Analysis

For Western blotting, 10 μg of protein lysate per sample was resolved by SDS-PAGE (10% gel) and transferred onto nitrocellulose membranes. Membranes were incubated with primary antibodies listed in Table 1, followed by peroxidase-conjugated secondary antibody (P0217, Dako). Signals were visualized with Fusion Solo S imaging system (M&S Instruments Inc., Osaka, Japan).

For dot blotting, lysates (1 μg/10 µL TBSB) were applied to nitrocellulose using a Bio-Dot SF Microfiltration Apparatus (#1706542, Bio-Rad, Tokyo, Japan), dried, and processed similarly to Western blots.

### 4.13. Knockdown Assay

CKB and MTCK1 were knocked down using siRNA (siCKB and siMTCK1, respectively; Santa Cruz Biotechnology Inc., Santa Cruz, CA, USA); control cells were transfected with non-targeting siRNA (siC). siRNA (50 nM for 2 × 10^5^ cells) was diluted in transfection solution according to manufacturer’s instructions. Gene expression was analyzed 48 h post-transfection.

### 4.14. Animals

Four-week-old BALB/c Slc-nu/nu mice were purchased from SLC Japan Inc. (Shizuoka, Japan). The animals were maintained and subjected to experiments in accordance with the institutional guidelines approved by the Committee for Animal Experimentation of Nara Medical University and the current regulations and standards of the Ministry of Health, Labour, and Welfare of Japan (approval nos. 12807, 28 May 2020, and 13093, 20 June 2021). Animals were acclimated to their housing for 7 days before the start of the experiment.

### 4.15. Animal Tumor Models

For the tumorigenesis assessment, SaOS2 cells (1 × 10^7^ cells/mouse), pretreated with siCK (siCKB + siMTCK1) or siC, were injected subcutaneously into nude mice (*n* = 10 per group). Tumor formation and growth were evaluated 4 weeks post-inoculation.

For local siRNA delivery, SaOS2 xenografts were established (1 × 10^7^ cells/mouse), and after tumors reached 5 mm diameter, liposomes containing siCK or siC (50 nM, 50 μL total volume) were injected intratumorally twice weekly using a 27-gauge needle.

For lung colonization experiments, SaOS2 cells were labeled with PKH26 (Sigma-Aldrich) and injected via tail vein (1 × 10^6^ cells/mouse). At 12 h, circulating tumor cells (CTCs) were isolated by dextran sedimentation and expanded in culture for colony counting. At 2 weeks, lungs were harvested for fluorescence imaging using the IVIS Spectrum system (Summit Pharmaceutical International, Tokyo, Japan). Group size calculations were performed using EZR software (v4.3.1, Jichi University Hospital).

### 4.16. Statistical Analysis

Statistical significance was performed using ordinary one-way ANOVA with Bonferroni correction (InStat 3.1, GraphPad, Los Angeles, CA, USA). Data are presented as mean ± SD. A two-sided *p*-value < 0.05 was considered to indicate statistical significance.

## 5. Conclusions

**Figure 8:**
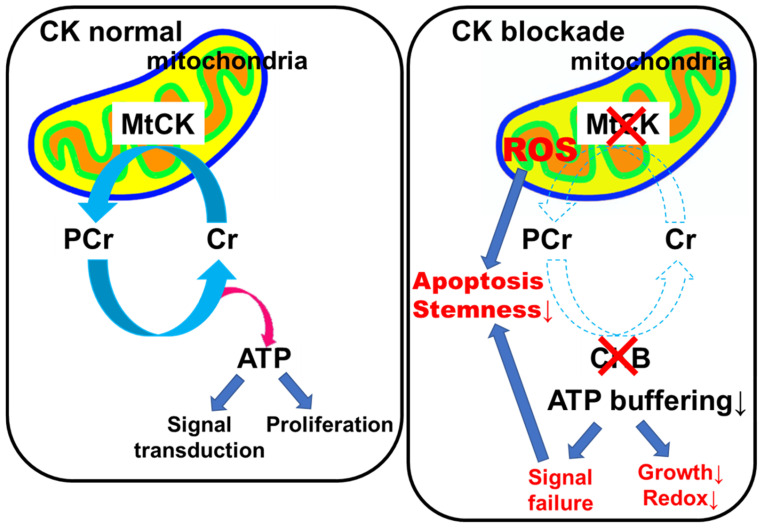
Effects of CK blockade. Creatine kinase (CK) blockade disables the phosphocreatine (pCr) energy shuttle in cancer cells, leading to ATP-buffering failure, mitochondrial/oxidative stress, redox impairment, apoptosis, and reduced stemness. In vivo, CK inhibition restrains tumor growth and dissemination. MtCK, mitochondrial CK; Cr, creatine; ROS, reactive oxygen species; Red X symbol, inhibition.

Creatine kinase (CK) blockade disables the phosphocreatine (pCr) energy shuttle in cancer cells, leading to ATP-buffering failure, mitochondrial and oxidative stress, apoptosis, and reduced stemness and motility. In vivo, CK inhibition suppressed tumor growth and dissemination, supporting CK axis disruption as an actionable metabolic vulnerability in osteosarcoma (Figure 8).

## Figures and Tables

**Table 1:** PCR primers, antibodies, siRNAs, and ELISA kits.

PCR Primers			
Gene	ID	Forward	Reverse
*human* Oct3	BC117437.1	gaaggatgtggtccgagtgt	gtgaagtgagggctcccata
*human nestin*	NM_006617.1	aacagcgacggaggtctcta	ttctcttgtcccgcagactt
*human CKB*	NM_001823.4	catatcaagctgcccaacct	accagctccacctctgagaa
*human mtCK*	J05401.1	gccgctactacaagctgtcc	cctggtgtgatcctcctcat
*human ALPL*	AH005272.2	ccagggaaatctgtgggcat	ccctaccttccaccagcaag
**Antibody (clone)**	**Cat No.**	**Company**	
pS (A4A)	05-1000	Merck, Darmstadt, Germany	
pT (PTR-8)	P6623	Merck, Darmstadt, Germany	
pY (4G10)	05-1050	Merck, Darmstadt, Germany	
human CKB	18713-1-AP	Proteintech, Rosemont, IL, USA	
human MtCK1	15346-1-AP	Proteintech, Rosemont, IL, USA	
human MtCK2	13207-1-AP	Proteintech, Rosemont, IL, USA	
human β-actin	JAN4548995073129	Fuji Film WAKO, Osaka, Japan	
human AKT	#9272	Cell Signaling Technology, Danvers, MA, USA	
human pAKT, Ser473 (D9E)	#11861	Cell Signaling Technology, Danvers, MA, USA	
human ERKp42	#9108	Cell Signaling Technology, Danvers, MA, USA	
human pERKp42, Tyr204 (E4)	sc-7383	Santa Cruz Biotechnology, Dallas, TX, USA	
human STAT3 (124H6)	#2217	Cell Signaling Technology, Danvers, MA, USA	
human pSTAT3, Tyr705 (B7)	sc-8059	Santa Cruz Biotechnology, Dallas, TX, USA	
human Ki67	ab15580	Abcam, Waltham, MA, USA	
**Small interfering RNA**	**Cat No.**	**Company**	
siC (Stealth RNAi)	12935-300	Thermo Fisher, Tokyo, Japan	
siCKM	abx901083	Abbexa, Cambridge, UK	
siMTCK1	abx911914	Abbexa, Cambridge, UK	
siMTCK2	abx911918	Abbexa, Cambridge, UK	
**ELISA**			
**Target**	**Cat No.**	**Company**	
human AMPK	MBS2514316	MyBioSource, San Diego, CA, USA	
human PKM2	NBP3-18036	Novus Biologicals, Centennial, CO, USA	
human AKT1/2/3	ab253299	Abcam, Waltham, MA, USA	
human PFK2	#SG-00103	Sinogeneclon, Hangzhou, China	
CK activity	MAK116	Merck, Darmstadt, Germany	
mouse Ki-67	#14507	Cell Signaling Technology, Danvers, MA, USA	
ATP	ab83355	Abcam, Waltham, MA, USA	
NADPH	ABIN771004	antibodies-online, Limerick, PA, USA	
4HNE	ab287803	Abcam, Waltham, MA, USA	
GSH	CEA294Ge	CLOUD-CLONE, Wuhan, China	
pCr	ELK8254	ELK Biotechnology, Sugar Land, TX, USA	
Lactate	ab65331	Abcam, Waltham, MA, USA	

ELISA, enzyme-linked immunosorbent assay; oct3, POU Class 5 Homeobox 1; CKB, creatine kinase B; mtCK, mitochondrial creatinine kinase; ALPL, alkaline phosphatase, liver, bone, kidney isotype; pS, phosphoserine; pT, phosphothereonine; pY, phosphotyrosine; pAKT, phosphorylated AKT; ERK, extracellular signal-regulated kinase; pERK, phosphorylated ERK; STAT, signal transducers and activator of transcription; pSTAT3, phosphorylated STAT3; si, short interferring RNA; siC, negative control siRNA; AMPKα1, 5′-AMP-Activated Protein Kinase Catalytic Subunit Alpha-1; PKM2, pyruvate kinase M2; PFKFB2, 6-Phosphofructo-2-Kinase/Fructose-2,6-Bisphosphatase 2; CK, creatinine kinase; NADPH, nicotinamide adenine dinucleotide phosphate; ATP, adenosine triphosphate; 4HNE, 4-hydroxynonenal; GSH, glutathione; pCr, phospho-creatine.

## Data Availability

Data is contained within the article.

## Supplementary Materials

The following supporting information can be downloaded at: https://www.mdpi.com/article/10.3390/ijms262311555/s1.

## Abbreviations

CK	creatinine kinase
CKB	creatinine kinase
MTCK	mitochondrial creatinine kinase
DNFB	dinitrofluorobenzene
OS	osteosarcoma
pCr	phosphocreatine
ROS	reactive oxygen species
CyC	cyclocreatine
KD	knockdown
VDAC	voltage-dependent anion channel
oct3	POU Class 5 Homeobox 1
pS	Phosphoserine
pT	Phosphothereonine
pY	phosphotyrosine
si	small interfering RNA
siC	negative control siRNA
AMPKα1	5′-AMP-Activated Protein Kinase Catalytic Subunit Alpha-1
PKM2	pyruvate kinase M2
PFKFB2	6-Phosphofructo-2-Kinase/Fructose-2,6-Bisphosphatase 2
NADPH	nicotinamide adenine dinucleotide phosphate
ATP	adenosine triphosphate
4HNE	4-hydroxynonenal
GSH	glutathione
