# Supplementary material for: Creatine Kinase Blockade Disrupts Energy Metabolism and Redox Homeostasis to Suppress Osteosarcoma Progression
Source: Int J Mol Sci. 2025 Nov 28;26(23):11555. doi: 10.3390/ijms262311555 (PMC12692347; doi:10.3390/ijms262311555)
Supplement: Supplementary file 1 [file ijms-26-11555-s001.zip › ijms-3987689-supplementary.pdf]

Supplementary Figure S1

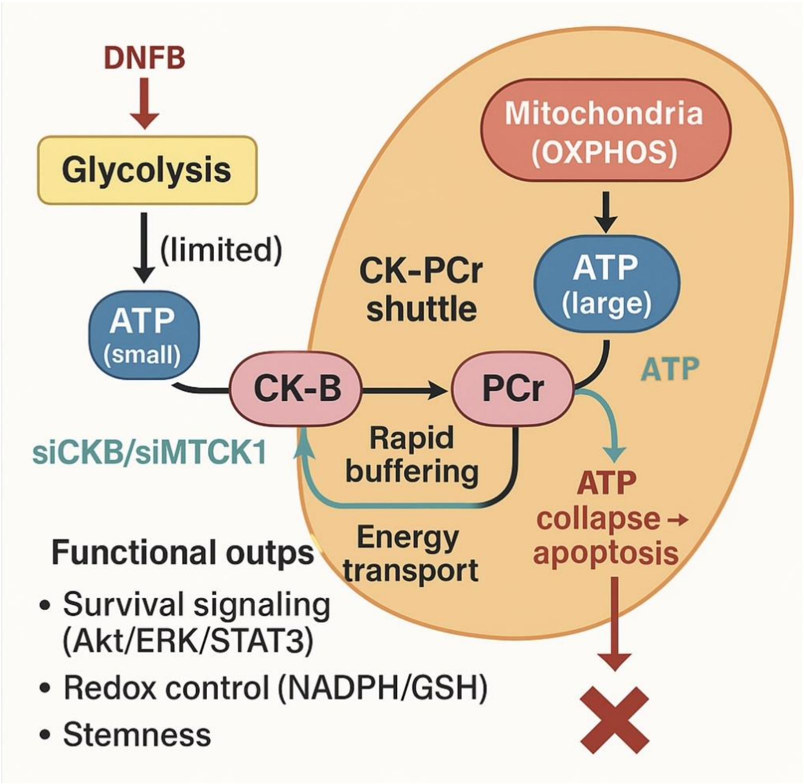

Supplementary Figure S1. Schematic overview of the creatine kinase (CK)–phosphocreatine (PCr) shuttle and its disruption by CK inhibition. Cancer cells maintain ATP homeostasis through the CK–PCr energy-buffering system, which links glycolytic ATP production with mitochondrial oxidative phosphorylation. Mitochondrial CK (CK-MT) converts ATP generated by OXPHOS into PCr, which diffuses to the cytosol and is rapidly reconverted to ATP by cytosolic CK-B at sites of high energy demand. This shuttle stabilizes survival signaling pathways (Akt, ERK, STAT3), redox balance (NADPH/GSH), and stemness. Pharmacological inhibition with DNFB or genetic suppression by siCKB/siMTCK1 disrupts this circuit, leading to ATP collapse, impaired phosphorylation signaling, and apoptosis. The diagram summarizes the energetic and functional consequences of CK inhibition in osteosarcoma cells.

Supplementary Figure S2

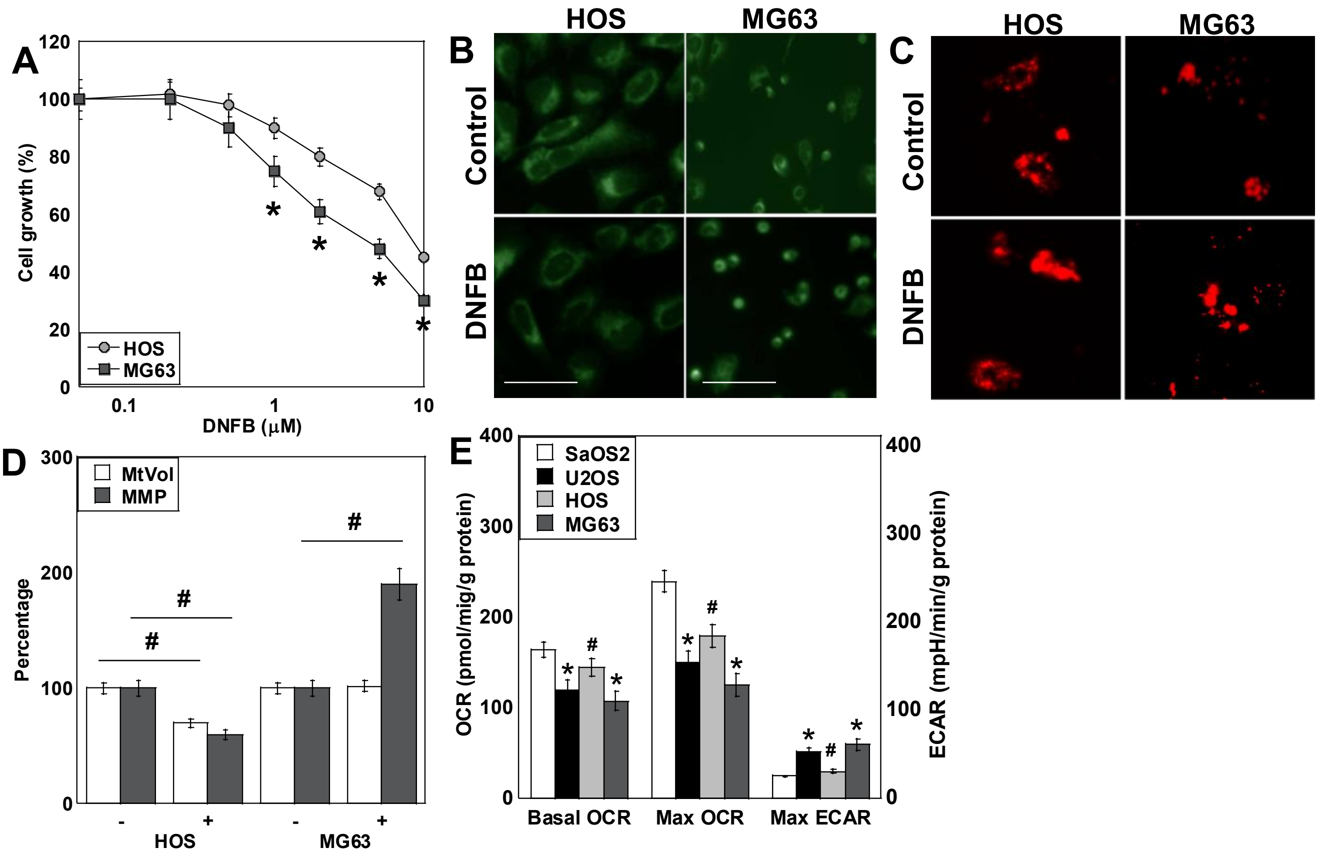

Supplementary Figure S2. Effects of DNFB on mitochondrial function and metabolic profiles in osteosarcoma cells.

(A) Cell viability of HOS and MG63 cells treated with DNFB (0.1–10 μM, 48 h) assessed by MTS assay. Data represent % viability relative to vehicle (0.1% DMSO). **(B)** Mitochondrial mass assessed by MitoTracker Green (50 nM, 30 min). Representative fluorescence images with scale bars (50 μm). **(C)** Mitochondrial membrane potential (MMP), detected with TMRE (50 nM, 30 min). Representative images, scale bars (50 μm). **(D)** Quantification of mitochondrial volume (MitoTracker) and MMP (TMRE). Data shown as % of control. **(E)** Basal and maximal oxygen consumption rate (OCR) and maximal ECAR in SaOS2, U2OS, HOS, and MG63 cells measured by Seahorse XF Analyzer. OCR/ECAR normalized to total protein. Statistical significance indicated as *$p < 0.05$ vs control; #$p < 0.05$ between cell lines. Error bars: standard deviation of ten mice. Statistical differences were calculated using ordinary ANOVA with Bonferroni correction.

ANOVA, analysis of variance; DNFB, dinitrofluorobenzene; OCR, oxygen consumption ratio; ECAR, extracellular acidification rate; MtVol, mitochondrial volume; MMP, mitochondrial potential.

Supplementary Figure S3

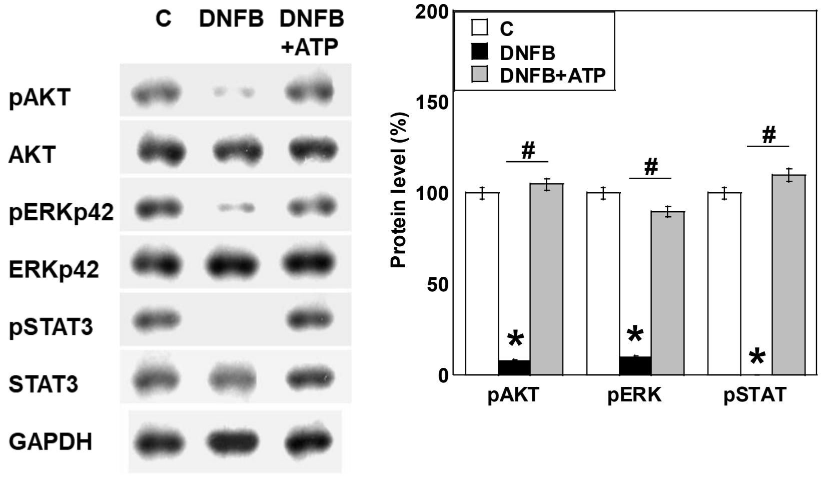

Supplementary Figure S3. DNFB suppresses phosphorylation of Akt, ERK, and STAT3, and ATP restores signaling.

SaOS2 cells were treated with DNFB (10 μM, 6 h) with or without extracellular ATP (2 mM, final 1 h). **Left panel:** Representative Western blots for p-Akt (Ser473), Akt, p-ERK1/2, ERK1/2, p-STAT3 (Tyr705), STAT3, and GAPDH. **Right panel:** Densitometric quantification of phosphorylated/total protein ratios normalized to control (%). DNFB markedly reduced phosphorylation of all three kinases, and ATP partially rescued these decreases. *$p < 0.05$ vs control; #$p < 0.05$ vs DNFB alone. Error bars: standard deviation of three independent trials. Statistical differences were calculated using ordinary ANOVA with Bonferroni correction.

ANOVA, analysis of variance; DNFB, dinitrofluorobenzene; ERK, extracellular signal-regulated kinase; STAT, signal transducer and activator of transcription; pAKT, phosphorylated AKT; pERK, phosphorylated ERK; pSTAT3, phosphorylated STAT3, GAPDH, glyceraldehyde 3-phosphate dehydrogenase.

Supplementary Figure S4

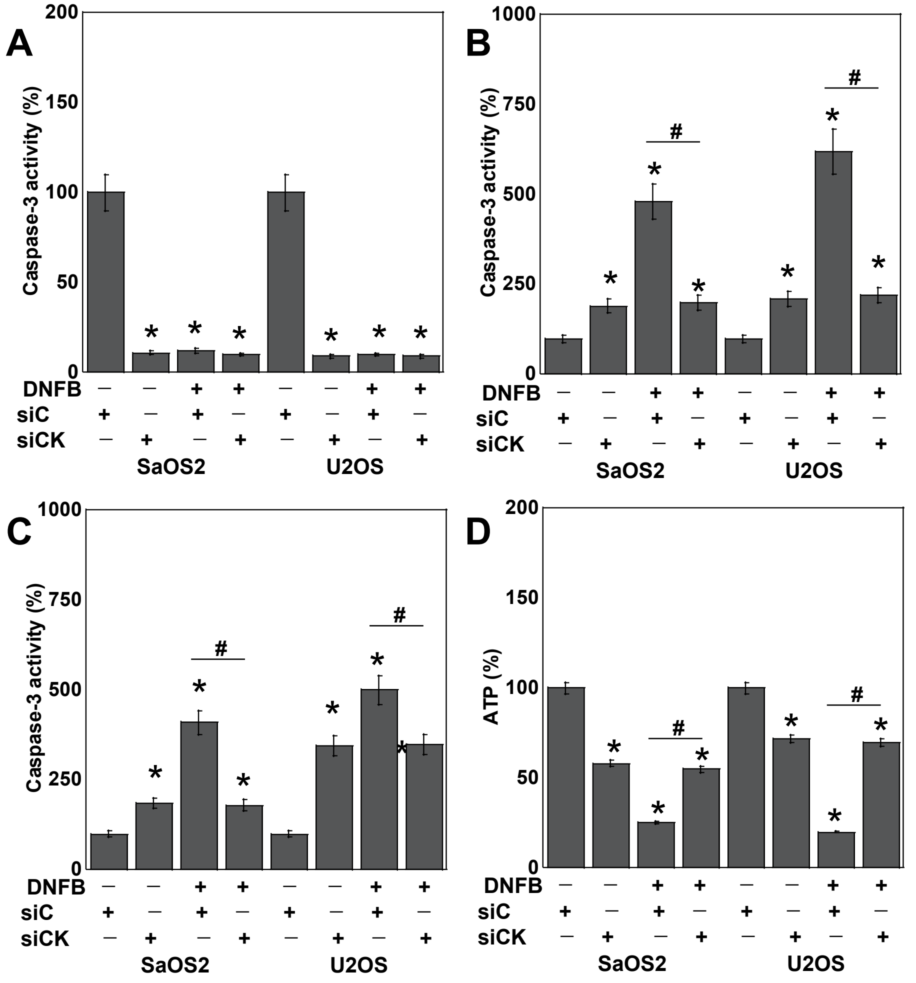

Supplementary Figure S4. Epistasis analysis combining CK knockdown and DNFB treatment in SaOS2 and U2OS cells.

Cells were transfected with control siRNA (siC) or dual CK knockdown (siCKB + siMTCK1; collectively siCK) for 24 h, followed by DNFB (10 μM, 24 h).

(A–C) Caspase-3 activity measured using fluorometric assay (excitation/emission 360/460 nm). Activity expressed as % of siC control.

(D) Intracellular ATP level measured by CellTiter-Glo. Values normalized to siC control.

CK knockdown increased caspase-3 activation and reduced ATP levels, and DNFB failed to further enhance these effects, indicating that DNFB cytotoxicity is largely mediated through CK inhibition. Statistics: *p < 0.05 vs siC; #p < 0.05 vs DNFB+siC. Error bars: standard deviation of three independent trials. Statistical differences were calculated using ordinary ANOVA with Bonferroni correction.

ANOVA, analysis of variance; DNFB, dinitrofluorobenzene; KD, knock-down; si, small interfering RNA; siC, control siRNA; siCKB, CKB siRNA; siMTCK, MTCK1 siRNA; MtMass, mitochondrial mass.

Supplementary Figure S5

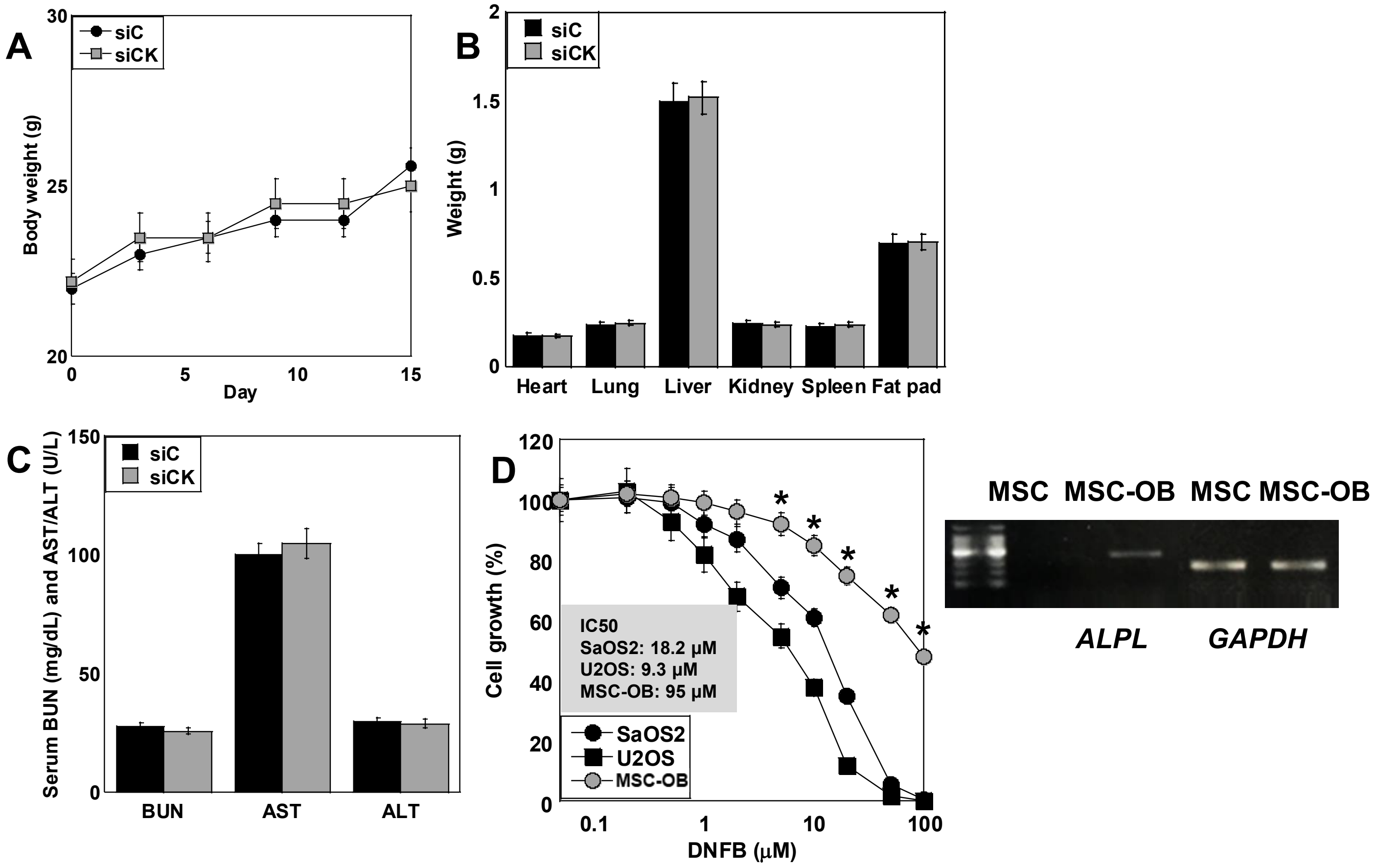

Supplementary Figure S5. Assessment of in vivo toxicity and DNFB sensitivity in normal osteoblast-like cells.

(A) Body weight of mice bearing SaOS2 xenografts treated with intratumoral siC or siCK (siCKB+siMTCK1) every 3 days. **(B)** Organ weights (heart, lung, liver, kidney, spleen, fat pad) collected at sacrifice (day 15). **(C)** Serum biochemical markers (BUN, AST, ALT) indicating systemic toxicity. No significant toxicity was observed in mice treated with CK knockdown. **(D)** DNFB cytotoxicity in SaOS2, U2OS, and human MSC-derived osteoblast-like cells (MSC-OB) treated with DNFB (0.1–100 μM, 48 h). IC_{50} values were: SaOS2 = 18.2 μM; U2OS = 9.3 μM; MSC-OB = 95 μM.**Right panel**: ALPL expression confirming osteoblastic differentiation in MSC-OB. GAPDH served as a loading control. *$p < 0.05$ vs. SaOS2 and U2OS. Error bars: standard deviation of ten mice. Statistical differences were calculated using ordinary ANOVA with Bonferroni correction.

ANOVA, analysis of variance; KD, knockdown; si, small interfering RNA; siC, control siRNA; siCK, siCKB+siMTCK1; BUN, blood urea nitrogen; AST, aspartate aminotransferase; ALT, alanine aminotransferase; MSC, human bone marrow mesenchymal stem cell; MSC-OB, MSC-derived osteoblastic cells; ALPL, alkaline phosphatase, liver, bone, kidney isotype, GAPDH, glyceraldehyde 3-phosphate dehydrogenase.
